# RNA-Seq reveals ACTH-induced steroid hormone pathway participating in goat adrenal gland response to castration

**DOI:** 10.1038/s41598-023-41016-5

**Published:** 2023-08-28

**Authors:** Haijing Jing, Yi Ding, Xunping Jiang, Guiqiong Liu, Yiyu Sha

**Affiliations:** 1 Key Laboratory of Agricultural Animal Genetics, Breeding and Reproduction of Ministry of Education, Huazhong Agricultural University, Wuhan, 430070 People’s Republic of China; 2 Laboratory of Sheep and Goat Genetics, Breeding and Reproduction, College of Animal Science and Technology, Huazhong Agricultural University, Wuhan, 430070 People’s Republic of China

**Keywords:** Transcriptomics, Vaccines

## Abstract

The content of androgen from adrenal is elevated under castration, and the mechanisms of compensatory secretion of adrenal androgen remain unknown. This study was designed to compare the transcript profiles between adrenals from noncastrated, orchiectomized and immunocastrated Yiling goats. Fifteen goats were randomly divided into three groups: pVAX-asd injection (control) group, pVAX-*B2L*-(G_4_S)_3_-kisspeptin-54-asd immunization (PBK-asd) group, and surgical castration (SC) group. Subsequently, serum was collected every two weeks after the initial immunization for hormone assays. At week 14 after immunization, adrenal glands were collected for transcriptome sequencing and qPCR. Serum testosterone concentration was significantly reduced in PBK-asd and SC group, demonstrating the effectiveness of castration. Both surgical and immunized castration resulted in adrenal hyperplasia, and thickness of adrenal cortex elevated. The specific genes involving castration were enriched in many pathways, including *Steroid hormone biosynthesis pathway*. The adrenocorticotropic hormone (ACTH), which promotes the production of adrenal steroids, and dehydroepiandrosterone (DHEA), a steroid hormone secreted by adrenal glands, both increased after castration. Further construction of co-expression network for transcription genes and traits (including adrenal weight and cortex thickness, ACTH and DHEA concentration) showed that the trait-related genes were enriched in multiple steroid-related pathways. These results showed that adrenal compensatory hyperplasia and androgen secretion caused by castration may involve in ACTH-induced steroid hormone synthesis.

## Introduction

Castration is a common management technique in livestock and poultry production to reduce aggressive behavior, improve carcass quality, and obtain higher economic benefits^1^. However, surgical castration is increasingly restricted due to animal welfare concerns. As a result, alternatives such as immunocastration have been developed. Immunocastration is a functional castration method that modifies the hormone balance of the hypothalamic-pituitary–gonadal axis (HPG axis) by reducing the secretion of gonadotropin-releasing hormone (GnRH), luteinizing hormone (LH), and follicle-stimulating hormone (FSH). This ultimately leads to a decrease in gonadal hormone levels, resulting in gonadal atrophy and functional inhibition^2^. Kisspeptin, produced by the KISS1 gene with its cognate receptor G protein-coupled receptor (GPR54), serves as an upstream hormone of the HPG axis^3^. Knockdown of KISS1 can cause gonadal atrophy in animals, making it a potential target for immunocastration^4^.

In male animals, androgens are secreted mainly in the testis and adrenal gland^5^. After castration, androgen secretion from testis is inhibited, resulting in a significant decrease in serum androgen levels. However, studies show that the adrenal gland becomes active and begins to secrete increasing amounts of androgens, playing a compensative role^6^. The adrenal gland is composed of about 80% cortex, which is divided into three layers: Zona reticularis (ZR), Zona fasciculata (ZF) and Zona glomerulosa (ZG). The ZG cells mainly secrete mineralocorticoid such as aldosterone while the ZF cells secrete glucocorticoids, primarily cortisol (corticosterone in rats). The ZR cells mainly secrete sex hormones like dehydroepiandrosterone (DHEA) and estradiol, and a small amount of glucocorticoids^7–9^. DHEA can be converted to testosterone in peripheral tissues, while testosterone can reduce the activity of the adrenal gland or inhibit the secretion of adrenal androgen^10^. When testosterone secretion is inhibited, adrenal gland may compensate by producing androgen precursor substances^6^. Several researches in rodents have shown that castration induces compensatory hyperplasia of adrenal cortex, enhancing the secretory activity of adrenal cortex cells, and thus can compensate for the lack of testosterone^11,12^. However, it has not been reported whether the adrenal glands of goat undergo similar changes in rodents after castration.

The hypothalamus–pituitary–adrenal (HPA) axis is a complex neuroendocrine system that regulates the secretion of adrenal corticosteroids^13^. In castrated adult rats, there is an increase in the levels of androstenedione, testosterone, and dihydrotestosterone, which are metabolized by DHEA from the adrenal glands increase^11^. Non-castrated males generally exhibit lower overall activity in the adrenal axis compared to castrated males, and castration can result in increased activity in the HPA axis^14,15^. The adrenal glands of castrated animals have an enhanced capacity to release corticosterone or cortisol^12,14,16,17^.

Transcriptome sequencing is a powerful tool for identifying differentially expressed genes associated to important economic traits of animals. It involves analyzing all RNA molecules transcribed by the cell, tissue, or organism under specific physiological or environmental conditions^18^. Functional annotation and enrichment analysis of these genes can provide insights into the molecular basis underlying various biological processes^19^. To date, only one study has investigated the modulatory effects of sex hormones on the adrenal transcriptome profile of gonadectomized rats. This study found that testosterone stimulated the expression of genes associated with lipids and cholesterol metabolism in male rats, while estradiol inhibited gene expression in intracellular signaling pathways^20^. As we know, no study has been conducted on the effects of castration on the transcriptome profile of the goat adrenal gland. Therefore, our study performed transcriptome sequencing on the adrenal glands of orchiectomized and immunocastrated Yiling goats to investigate specific adrenal genes involved in castration. The study analyzed the relationship between these genes and serum ACTH and DHEA levels, provided the first comprehensive transcriptional database of the adrenal gland following castration, hoping to shed light on the molecular mechanisms underlying this process.

## Results

### Effect of *KISS1* immunocastration

The serum anti-kisspeptin antibody and testosterone concentration in the experimental goats were presented in Table 1. At the second week after initial immunization, the PBK-asd group produced higher anti-kisspeptin antibody than the control and surgical castration groups (*p* < 0.05). The antibody titer reached its peak at 10 weeks and lasted until the 14 weeks after the initial immunized (*p* < 0.05). From the beginning to the end of the experiment, the serum testosterone concentration in the surgical castration group was significantly lower than the control group (*p* < 0.05). Compared with the control group, the serum testosterone concentration of the PBK-asd group was significantly decreased from 4 to 14 weeks (*p* < 0.05).

**Table 1:** The serum anti-kisspeptin antibody and testosterone concentration.

Week	Traits	Group		
		C	PBK-asd	SC
2	Anti-kisspeptin antibody (OD450)	0.18 ± 0.02^a^	0.70 ± 0.30^a^	0.16 ± 0.03^b^
4	Anti-kisspeptin antibody (OD450)	0.16 ± 0.04^a^	0.93 ± 0.26^b^	0.13 ± 0.03^a^
6	Anti-kisspeptin antibody (OD450)	0.12 ± 0.04^a^	1.75 ± 0.35^b^	0.04 ± 0.02^a^
10	Anti-kisspeptin antibody (OD450)	0.16 ± 0.04^a^	1.78 ± 0.21^b^	0.14 ± 0.08^a^
14	Anti-kisspeptin antibody (OD450)	0.14 ± 0.02^a^	1.12 ± 0.11^b^	0.15 ± 0.03^a^
2	Serum testosterone level (ng/ml)	1.50 ± 0.39^a^	1.27 ± 0.58^a^	0.03 ± 0.003^b^
4	Serum testosterone level (ng/ml)	1.86 ± 0.35^a^	1.01 ± 0.47^b^	0.03 ± 0.003^c^
6	Serum testosterone level (ng/ml)	2.07 ± 0.31^a^	0.38 ± 0.09^b^	0.03 ± 0.000^b^
10	Serum testosterone level (ng/ml)	2.33 ± 0.47^a^	0.53 ± 0.13^b^	0.03 ± 0.002^b^
14	Serum testosterone level (ng/ml)	2.57 ± 0.39^a^	0.96 ± 0.18^b^	0.026 ± 0.002^c^

The serum anti-kisspeptin antibody and testosterone concentration in control (n = 5), PBK-asd immunized (n = 5) and surgical castrated (n = 5) goats from 2 weeks after initial immunization to the end of the experiment at 14 weeks. All data are expressed as mean ± SE. Different superscript letter in the same row indicates a significant difference (*p* < 0.05).

### Adrenal section and cortical thickness

**Figure 1:**
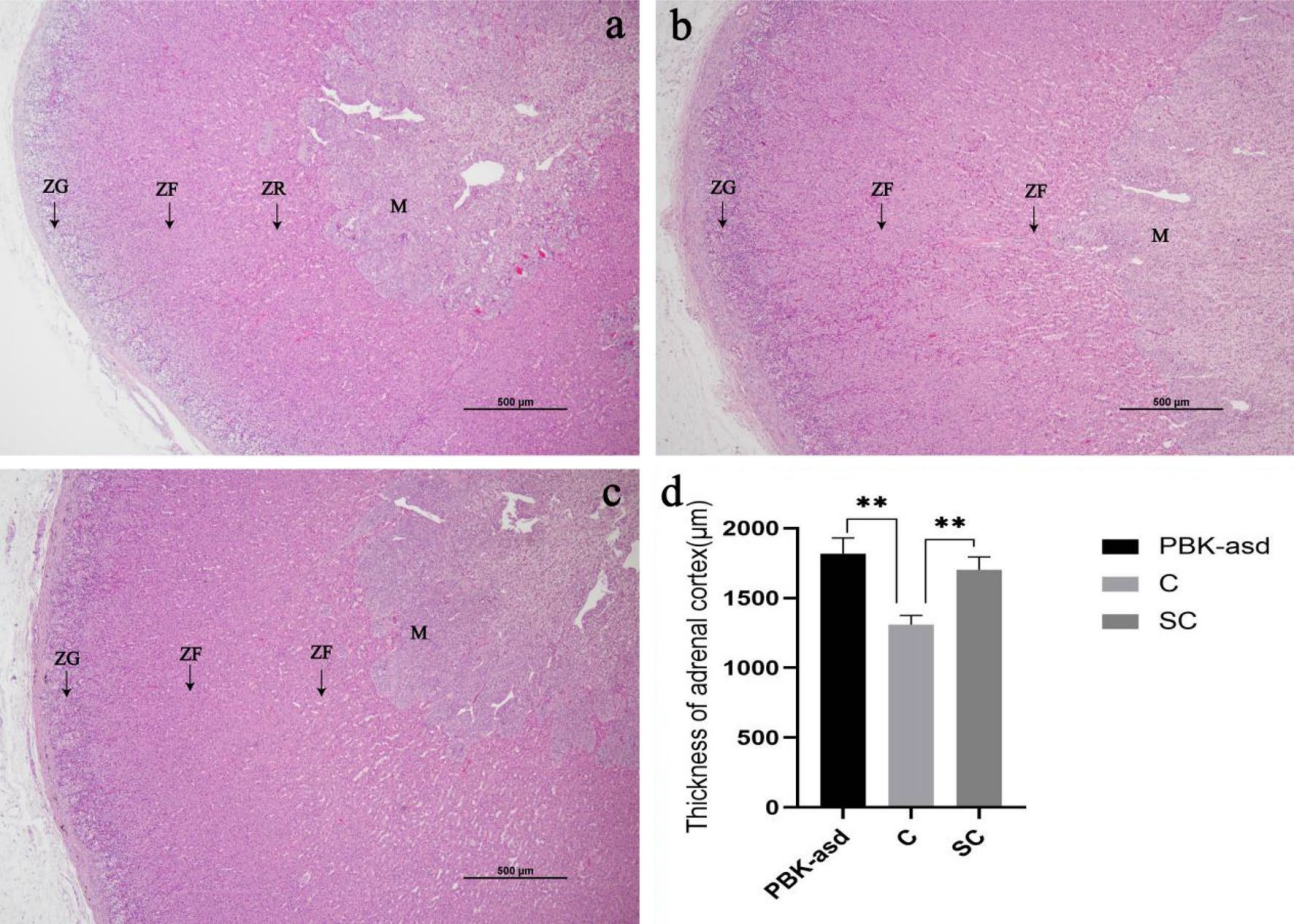
Adrenal section and cortical thickness. HE stains of adrenal gland. (**a**) is control group (C), (**b**) is immunocastration group (PBK), (**c**) is surgical castration group (SC), (**d**) is the adrenal cortical thickness in the three groups. All groups are observed and photographed under a 4X object lens. The pink part on the periphery is adrenal cortex, darker parts of the interior is adrenal medulla (M). In each panel, the part of cortex from left to right is the zona glomerulosa (ZG), zona fasciculata (ZF), and zona reticularis (ZR), respectively. All data are expressed as mean ± SE. Significance is indicated by ** (*p* < 0.01) vs control group.

Hematoxylin-eosin (HE) stains of goat adrenal showed very clear pink cortex and darker medulla, and clear ZG, ZF and ZR of cortex (Fig. 1). The ZG was relatively thin, and the cells were arranged into columnar and lumpy. The ZF was thick, and the cells were arranged in a cable shape. The thickness of ZR was between ZG and ZF, and the boundary between ZR cells and ZF was not obvious, but the boundary between ZG and ZF was clear. Compared with the control group (Fig. 1a), the adrenal cortex was thicker in the immunocastration (Fig. 1b) and surgical castration (Fig. 1c) groups. We randomly chosed several places of cortex in each group to measure its thickness and found that adrenal cortex thickness in the surgical and immune castration group were significantly higher than that in the control group (*p* < 0.01, Fig. 1D). No difference of cortex thickness was found between the two castration groups. Castration increased adrenal weight (*p* < 0.05), but there was no difference of adrenal weight between the two castration groups [adrenal weight (g): C 0.63 ± 0.01; PBK-asd 0.71 ± 0.03; SC 0.75 ± 0.02; in each group n = 5, mean ± SE].

### The expression profiles of differential genes in adrenal

**Figure 2:**
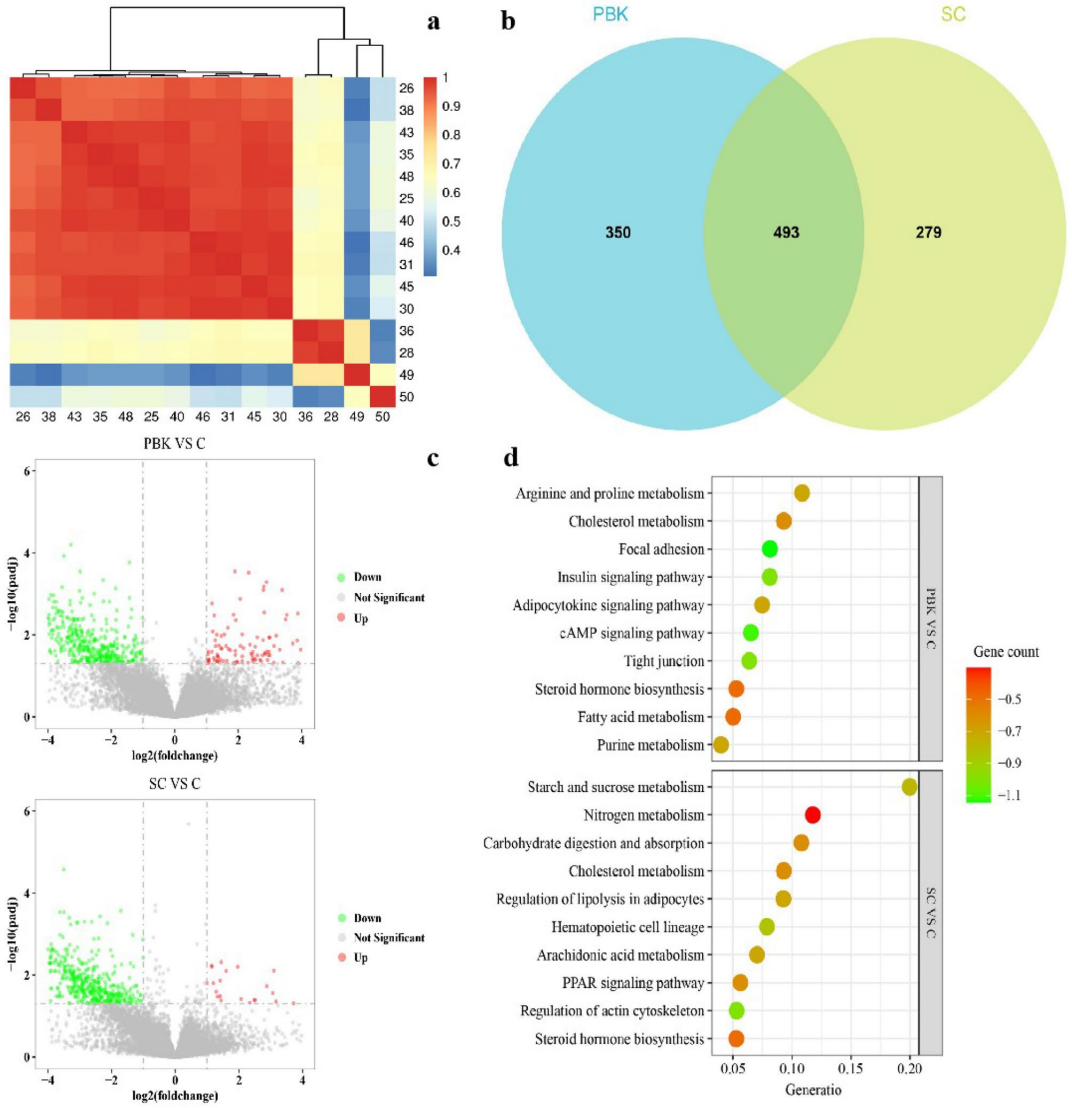
The expression profiles of differential genes in adrenal. Expression profiles of differential genes in adrenal. (**a**) Heat map of correlation coefficient for samples based on the gene expression level. Control group (**C**) 28, 36, 46, 49, 50; Surgical castration group (SC): 25, 31, 35, 45, 48; Immunocastration group (PBK): 26, 30, 38, 40, 43. (**b**) The Venn diagram for the numbers of DEGs in castration groups compared with control group; (**c**) Volcano map of DEGs in castration groups compared with control group. Red indicates the up gene while green indicates the down gene. (**d**) The bubble diagram with Gene number demonstrating the involved KEGG pathways. Generatio is the percent of DEGs to the whole genes in the pathway. Gene count is the number of the DEGs in the pathway.

The adrenal tissues of Yiling goats were sequenced for transcriptome analysis to investigate the expression profiles of genes. The correlation analysis demonstrated that the samples obtained from PBK and SC groups formed a distinctive cluster, the control group belonged to another separate cluster (Fig. 2a). Compared to the control group, the immunocastration group and the surgical castration group had 843 and 772 differentially expressed genes (DEGs), respectively, in which 493 DEGs overlapped (Fig. 2b). There were 158 up-regulated and 654 down-regulated DEGs in the immunocastration group while there were 30 up-regulated and 686 down-regulated DEGs in the surgical group (Fig. 2c). Kyoto Encyclopedia of Genes and Genomes (KEGG) pathway enrichment analysis was performed based on the overall differential genes of the SC group or PBK group (Fig. 2d). The result showed that the number of pathway terms from PBK versus C groups was 250 and that from SC versus C groups was 260, in which 237 terms overlapped, including steroid hormone related pathways, namely *Steroid hormone biosynthesis*, *Cholesterol metabolism*.

### Quantitative real-time PCR

**Figure 3:**
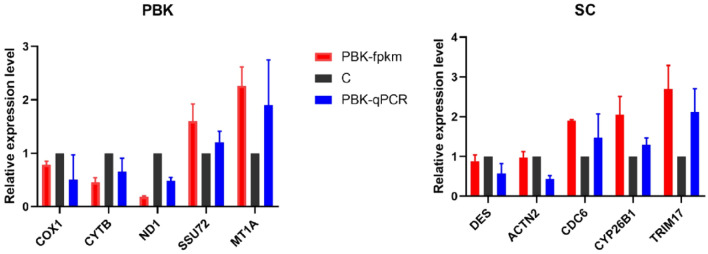
Quantitative real-time PCR. Validation of the RNA-seq results. PBK and SC show the specific gene expression level of RNA-seq and qPCR of PBK vs C and SC versus C groups. C: control group; SC: surgical castration group (SC); PBK: immunocastration group; fpkm: fragments per kilobase million mapped reads. Data are shown as means ± SE.

We randomly selected 5 DEGs in PBK and SC groups to verify the accuracy of the gene expression analysis. The results showed that the expression of these 10 DEGs in qRT-PCR was consistent with the results of transcriptome sequencing (Fig. 3), which proved the reliability of the high-throughput RNA-Seq data.

### Serum hormone of HPA axis

**Figure 4:**
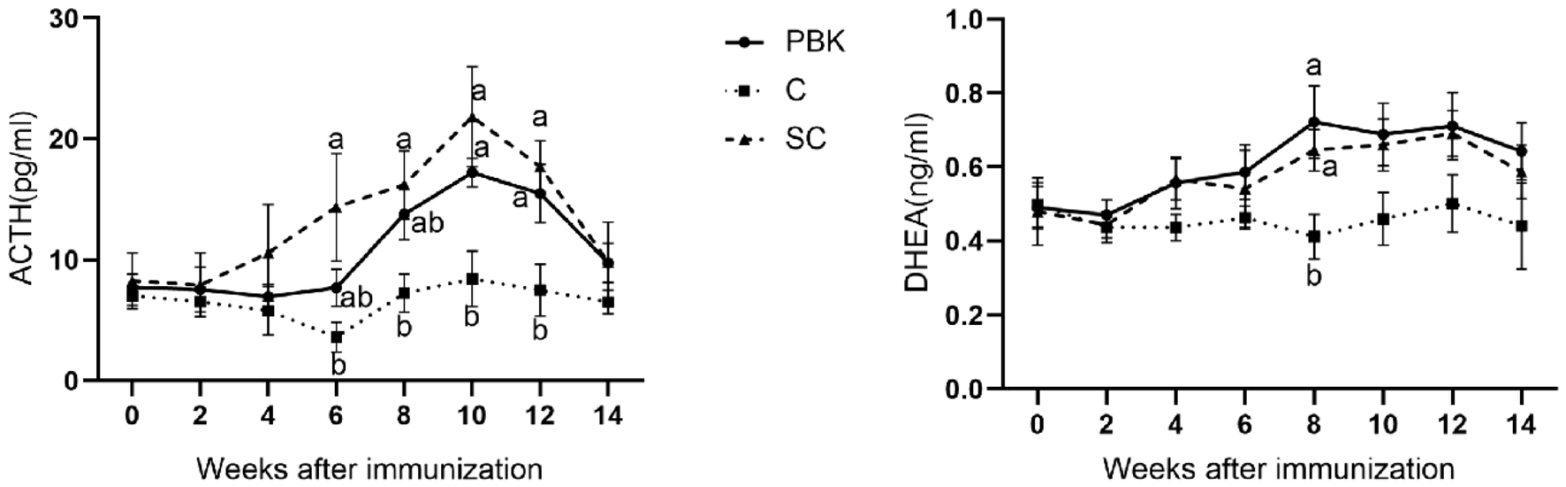
Serum hormone of HPA axis. Serum ACTH and DHEA levels in goats of the immunocastration group (PBK), the control group (C) and the surgical castration group (SC). All data are expressed as mean ± SE. Different letters at the same week indicate significant differences between the groups (*p* < 0.05).

The ACTH and DHEA hormones in the serum of goats were shown in Fig. 4. The ACTH level in the SC group was significantly higher than that of the control group from the 6th week to the end of the experiment (*p* < 0.05). And from the tenth week to the end of the experiment, the ACTH level in the PBK group was significantly higher than that of the control group (*p* < 0.05). The values of serum DHEA in SC and PBK groups were higher than that of the control group from the fourth week to the end of the experiment, but only the values at eighth week were significantly higher than that of the control group (*p* < 0.05). The results showed that both surgical castration and immunocastration resulted in the increment of ACTH secretion from pituitary gland and DHEA secretion from adrenal gland.

### Co-expression network construction and module mining

**Figure 5:**
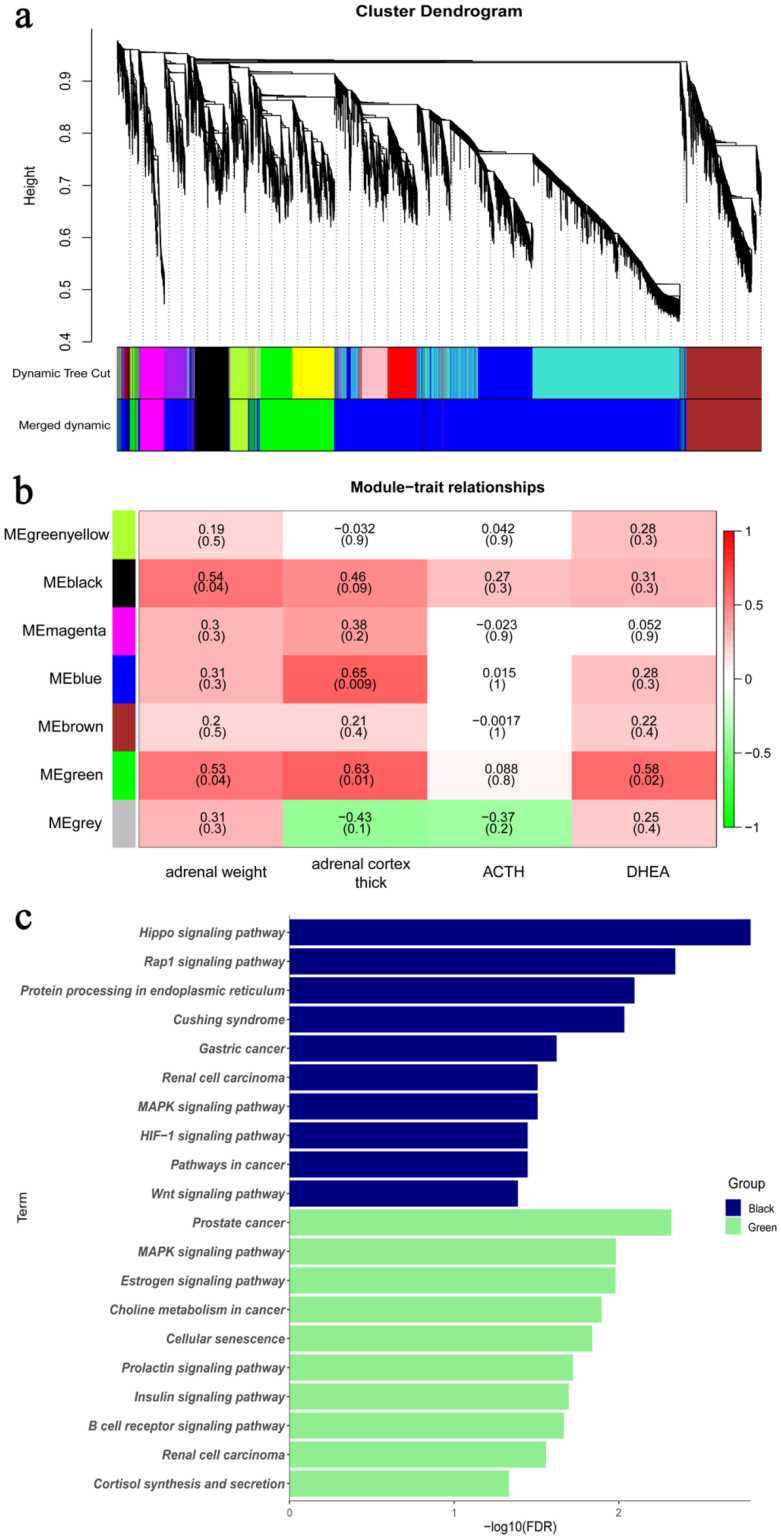
Co-expression network construction and module mining. Co-expression network construction and module mining. (**a**): Gene clustering tree based on hierarchical clustering. Modules corresponding to branches are labeled with colors indicate by the color bands underneath the tree. A total of six modules are identified; (**b**): Module-traits relationship. Each row corresponds to a module feature gene, and each column corresponds to a feature. Each cell contains the corresponding correlation and p-value; red indicates positive correlation and green indicates negative correlation; (**c**): The functional enrichment results for each module. The terms with the lower FDR are shown in the figure.

The weighted gene co-expression network analysis (WGCNA) method was used to cluster the expression genes after castration (Fig. 5a). The genes were clustered into six modules clustered by co-expression, and two modules were selected as the main modules (Fig. 5b,c). The black module was associated with the traits of adrenal, including adrenal weight, adrenal cortex thick, and the hormone secretion of ACTH and DHEA. The green module was also related to the adrenal weight, adrenal thick and secretion of DHEA. For black module (883 genes), the terms of KEGG pathway included *MAPK signaling pathway,* and *Wnt signaling pathway*, which is related to ACTH function*.* For green module (1677 genes), the enriched pathway included *Prostate cancer, MAPK signaling pathway, Estrogen signaling pathway, Choline metabolism in cancer, Prolactin signaling pathway, Insulin signaling pathway, Renal cell carcinoma* and *Cortisol synthesis and secretion*, all of which are related to ACTH and DHEA function.

## Discussion

In this study, it was observed that serum testosterone level decreased sharply after surgical castration. The kisspeptin antibody and testosterone concentration in serum can be used as indicators to evaluate the effectiveness of immunocastration. The serum testosterone concentration in goats significantly decreased after *KISS1* DNA vaccine injection, while the anti-kisspeptin antibody increased. These results proved the effectiveness of surgical castration and immunocastration. Previous researches on rodents have shown that castration can cause adrenal hyperplasia. A study conducted on Sahara gerbils during the breeding season demonstrated a significant increase in the height of adrenal cells, nucleus diameter, and overall activity of the adrenal axis in both surgically castrated and immunized gerbil groups compared to the control male gerbil group^21^. Similar results were observed in pigs, where the overall activity of the adrenal axis was higher in both surgically castrated and immunized male pigs compared to the control male pigs^15^. Our research on Yiling goats showed a significant thickening of the adrenal cortex in castrated goats, indicating adrenal compensatory hyperplasia in both surgically castrated and immunized goats.

Here, we aimed to investigate the pathways and genes involving in the changes induced by castration in the adrenal gland. Transcriptome analysis revealed that surgical castration and immunocastration resulted in 843 and 772 differentially genes, respectively, with 493 genes overlapping between the two methods. Subsequent functional annotation analysis identified several pathways, including *Steroid hormone biosynthesis* and *Cholesterol metabolism*, which were later found out to be related to adrenal function enhancement after castration. Steroid hormones are involved in many important physiological processes and play an important role in maintaining homeostasis^22^. All steroid hormones, including those synthesized by the adrenal cortex, are derived from cholesterol^23^. ACTH plays a crucial role in enhancing the steroidogenic capacity of adrenal cells by stimulating adrenal hypertrophy and hyperplasia^24^, which provides a cellular site for steroidogenesis^25^. After the release of ACTH from the pituitary gland, the intracellular cAMP level increases, leading to the activation of cAMP-dependent protein kinase (PKA) and ultimately resulting in the increased biosynthesis of steroid hormones^26,27^. ACTH acts primarily on the zona fasciculata and zona reticularis of the adrenal cortex to promote the synthesis of glucocorticoids and androgen precursors, respectively^28^. DHEA is the most abundant circulating steroid hormone secreted by the adrenal cortex and its production is regulated by ACTH^29^. Castration can result in adrenal hyperplasia due to the stimulation of ACTH, which leads to variation in DHEA concentrition. Based on the findings of transcriptome analysis above, we measured the concentration of steroid hormones in serum, including ACTH and DHEA, and found that both hormones increased following castration. The decrease in testicular androgen after castration may trigger a feedback response in the pituitary gland, resulting in increased secretion of ACTH. The abundant ACTH stimulates the proliferation of adrenal cortical cells, which produce pregnenolone through cholesterol side chain cleavage, mediated by the enzyme encoded by CYP11A1 (P450scc)^30^. Pregnenolone is further converted to 17-hydroxypregnenolone and then to DHEA under the action of microsomal P450C17 and 17,20-lyase of P450C17, respectively^31^. DHEA is a weak androgen secreted by the adrenal cortex and can be converted to testosterone in peripheral tissues^9^. Our findings suggested that the ACTH-mediated steroidogenesis pathway mag be involved in enhancing adrenal secretion after castration. This indicates that the HPA axis activity is enhanced, and the adrenal gland secretes more androgen as a compensatory response to castration.

Castration in male rats may lead to a decrease in inflammatory responses, possibly due to compensatory secretion of glucocorticoids and the inhibition of COX-2 expression^32^. COX-2 isoenzyme is localized in the PVN of the hypothalamus, which plays a role in modulating the response of CRH to HPA axis^33^. The HPA axis is mainly controlled by neurons in the PVN, which releases CRH and drives ACTH release from the anterior pituitary^34,35^. Glucocorticoids secreted by the adrenal cortex have anti-inflammatory effects, and DHEA has also been shown to attenuate inflammatory responses^36–38^. This study suggests that castration may lead to a decrease in inflammatory response in male rats. This could be attributed to an increase in compensatory secretion of glucocorticoids after castration, which can counteract the effect of some inflammatory factors and inhibit the expression of COX-2. Additionally, binding of ACTH to its receptors can promote activation of signaling cascades that initiate adrenal-specific steroidogenic transcriptional programs, which may explain the increment in ACTH-controlled steroidogenic enzymes after castration^39^. Overall, these findings suggest a potential association among the HPA axis, inflammation, and castration in male rats. Binding of ACTH to its receptors leads to the activation of PKA and MAPK-dependent signaling cascades that collaborate to initiate adrenal-specific steroidogenic transcriptional programs. This process ultimately leads to glucocorticoids synthesis and secretion from the adrenal cortex^40^. After castration, there is an increase in ACTH secretion and a high expression of ACTH-controlled steroidogenic enzymes. *COX-2* gene expression is inhibited by ACTH-mediated steroid hormone biosynthesis. *COX-2* may be involved in the negative regulation of steroidogenesis by MAPK because the expression of *COX-2* was down-regulated in adrenal tissues after castration^41^. The activation of COX-2 signaling pathway may involve in the biosynthesis of steroid hormones mediated by ACTH. Thus, these two pathways may cooperate to participate in the compensatory process of the adrenal gland.

In this paper, we found that several genes of the Wnt family existing in co-expression network modules. Canonical Wnt signaling plays a crucial role in maintaining the homeostasis of the adrenal cortex and is involved in the differentiation of progenitor cells in a variety of organ systems^42^. The adrenal hypertrophy caused by castration in male mice is a result of increased production of corticosteroid^43^. During times of higher physiological demand for steroid production, endocrine signals transcend paracrine inputs and promote differentiation and steroidogenesis^44^. It has been demonstrated that cAMP/PKA inhibits canonical Wnt signaling pathway^45^. The ACTH signaling pathway antagonizes canonical Wnt signaling in the adrenal cortex. Canonical Wnt signaling can also be suppressed by noncanonical Wnt signaling and has been associated to adrenal steroid hormone production^46^. Therefore, it can be hypothesized that noncanonical Wnt signaling plays a synergistic role with ACTH in the production of adrenal steroid hormones. Based on these findings and our RNA-seq results, we hold the opinion that the adrenal ACTH signaling pathway is enhanced after castration, and this pathway collaborates with COX-2 signaling pathway and Wnt signaling pathway to stimulate steroid hormone secretion and participate in the compensatory secretion of adrenal androgen.

## Conclusion

Our results showed that both immunocastration and surgical castration can cause adrenal hyperplasia and compensatory androgen secretion in goat, and the potential reason for this may be the up-regulation of ACTH-induced steroid hormone pathway.

## Materials and methods

### Ethics statement

The study was carried out in accordance with the guidelines for the care and use of experimental animals for the scientific purpose set by the Ministry of Science and Technology (Beijing China, No.398, 2006). The institutional animal care and use ethics committee of Huazhong Agricultural University approved the protocol (HZAUGO-2019-006). All methods are reported in accordance with ARRIVE guidelines.

### Vaccines and animals

The immunocastration vaccine pVAX-*B2L*-(G_4_S)_3_-kisspeptin-54-asd (PBK-asd) and empty plasmid vaccine (pVAX-asd) were previously constructed by Wassie et al.^47^ The PBK-asd is a recombinant kisspeptin-54 and *B2L* vaccine, which is an effective immunocastration vaccine, can inhibit testicular development and animal sexual behavior. The 15 healthy male Yiling goat (5 month, 17 ± 1.8 kg) were provided by Yichang Bailihuang Husbandry Co. Ltd (Yichang, Hubei, China, five goats each group). All the goats were sheltered in the same house with similar environmental conditions. All goats were in the spermatogenesis stage throughout the experiment. They were provided ad libitum water and fed 3–4 kg/day silage and peanut vine in ratio of 6: 4 supplemented with concentrate (1% of their body weight) containing 64% corn, 20% soya bean, 8% rice bran, 5% premix, 1.5% salt, and 1.5% soda ash.

### Vaccine immunization and sample collection

The goats were randomly divided into three groups: pVAX-asd injection (control group), PBK-asd immunization and surgical castration. Surgical castration was performed two weeks before the plasmid injection. The goats in the immunized treatment and control group were injected intramuscularly with PBK-asd and pVAX-asd plasmid (1 mg/dose), respectively. Both PBK-asd and control group goats received a two-booster immunization with 3-week intervals.

Blood samples were collected from the jugular vein every two weeks from day 0 of immunization to the end of the experiment (lasting 14 weeks). Blood samples (5 ml) were collected using a coagulant collecting vessel (Aosaite, Shandong, China) which were stored at 4 °C overnight, separated for serum by centrifuging at 3000 rpm for 10 min at 4 °C and stored at −20 °C. Serum was used for hormone assays by ELISA. The goats were anesthetized at 14 weeks after the initial immunization by intramuscular injection with 0.1 ml/kg xylazine hydrochloride (Shengda, Changchun, Jilin, China), and all efforts were made to minimize suffering. Both adrenal glands were collected from each goat, the left adrenal gland was fixed with 4% paraformaldehyde, the right adrenal gland was immediately frozen in liquid nitrogen and then stored at −80 °C. Half of the collected adrenal gland was frozen in liquid nitrogen to extract RNA for transcriptome sequencing and fluorescence quantification, and the another half was fixed for H&E analysis.

### Hematoxylin–eosin stains of adrenal gland

The adrenal was fixed with 4% paraformaldehyde solution for 2 weeks, washed with water, dehydrated using graded ethanol, vitrified by dimethylbenzene and deposited in holly oil. The tissues were sliced into sections of 4 µm thick and stained with HE. Each adrenal section was observed with Eclipse-Ci™ microscope (NIKON, Chiyoda, Tokyo, Japan). Image Pro Plus 6.0 was used to trace the ZG band edges and ZR-medulla boundaries throughout the section and the average thickness was measured.

### Library construction and RNA sequencing

Total RNA was reverse-transcribed using the PrimeScript RT reagent Kit with gDNA Eraser (Toyobo, Osaka, Japan). The cDNA was amplified and purified with Qubit dsDNA high-sensitivity kit, then the fragments of approximately 200 bp in length were selected for library construction with NEBNext Ultra RNA library preparation kit. The libraries were paired-end sequenced using the Illumina HiSeq X Ten platform from Megagenomics Company (Beijing, China).

### Sequencing data filtering

The Raw data obtained by sequencing were filtered, and Clean Reads were obtained by removing connectors and low-quality Reads. Clean reads were aligned with reference genome sequences using Hisat2 software for transcript assembly. FPKM (Fragments Per Kilobases of transcript per Million mapped reads) was used to homogenize gene expression.

### Differential gene expression analysis

The differentially expressed genes (DEGs) were calculated using the DESeq2 package^48^. We compared the control samples with immunocastration group or surgical castration group to obtain the DEGs. The P value for each gene was obtained based on the model of negative binomial distribution. The fold changes were estimated using the DESeq2 package. The screening criteria for DEGs were *p* < 0.05 and |fold change|≥ 2. Then the specific DEGs were combined to one DEG union set for the subsequent analysis.

### Determination of serum hormones

Testosterone, anti-kisspeptin, ACTH and DHEA were measured by enzyme-linked immunoassay (ELISA) kits (Huaying Bioengineering Co. Ltd., Beijing, China) as described in the instructions. The detection range of testosterone was 0.06–2.88 ng/ml, the sensitivity was 1.0U/L for anti-kisspeptin. The ACTH and DHEA hormone levels were measured using antibody against goat ACTH, DHEA. The detection range of ACTH and DHEA were 3–85 ng/L and 0.32–17.62 ng/ml. All inter- and intra-assay coefficients of variation (CV) were less than 10%.

### Weighted correlation network analysis

Gene co-expression networks were constructed using the R package weighted correlation network analysis (WGCNA)^49^. The Fragments Per Kilobase of exon model per Million mapped fragments (FPKM) values of all grouped genes were inputted for analysis. To determine the functions of the genes in the trait-associated modules, genes were subjected to web server KOBAS (http://kobas.cbi.pku.edu.cn/kobas3/genelist/) for pathway enrichment. *p* < 0.05 was the threshold used for the significant terms.

### Quantitative real-time PCR

The cDNA was diluted 10 times and then used as the template of qRT-PCR. The reaction system contained 5 μl of 2 × Hieff® qPCR SYBR Green Master Mix (Yeasen Biotech, Shanghai, China), 1 μl of cDNA, 0.5 μl of upstream primer, 0.5 μl of downstream primer and deionized water in a final volume of 10 μl. The PCR was conducted at 94 °C for 5 min, followed by 35 cycles of 94 °C for 10 s, 55 °C for 20 s and 72 °C for 6 s, and 5 min extension. The gene expression level was determined by the 2^-ΔΔCt^ algorithm, and the goat *β-actin* gene was used as an internal control^50^. Each sample had three biological replicates, and the gene expression level was presented as the means ± standard errors (SE) (n = 3). The primer sequences of the selected genes were shown in Table 2.

**Table 2:** Primer sequences for qRT-PCR test.

Gene	Forward primer (5′-3′)	Reverse primer (5′-3′)	Product length (bp)	Gene bank accession number
ACTB^a^	gtcaccaactgggacgacat	catcttctcacggttggcct	132	NM_001314342.1
COX1	acgtcgatacacgggcttac	ctcatatcatggcgggggac	129	NC_005044
CYTB	caaagccactctcacccgat	tgtggggttgttcgatcctg	115	NC_005044.2
ND1	cctacccataccctaccccc	tgtgctactgctcgtaaggc	150	NC_005044.2
SSU72	ctgccagtgtatccagcaca	agtagaagcagacggtgtgc	110	NC_030823.1
MT1A	atggacccgaactgctcct	agcagctcttcttgcaggag	100	XM_005692001
DES	gcgcaggatcgaatctctca	gctgtgaggtctggtttcga	138	XM_013972881
ACTN2	cttgatcactgcgcatgagc	gggttgcttgagctgatcct	135	XM_018042499
CDC6	cgtctcggacagtggacaaa	ggttgtcatcacccagacgt	135	NC_030826.1
CYP26B1	tgaccatgcaggagctgaag	cagcagctgcatgataagcg	98	NC_030818
TRIM17	acagcctgagtgtgcagtac	ggcaccacatcctcttggaa	95	NC_030814.1

^a^Gene ACTB was used as an internal control.

### Data analysis

Data were analyzed using the software program IBM SPSS Statistics (Version20). The normality of the variance of the data were checked using Shapiro–Wilk test. The data of antibody and hormone concentration were analyzed by One-way ANOVA and Tukey’s multiple comparison post-hoc tests. The relative quantitative method was used for gene expression analysis, the control group was default to 1, and the results of the treatment group were measured by their ratio to the control group. All data were expressed as mean ± SE, and statistically significant was considered when *p* < 0.05.

## Data Availability

The datasets presented in this study can be found in online repositories. The data is available under the accession number PRJNA885017 from NCBI database.

## Abbreviations

HPA: Hypothalamic–pituitary–adrenal
ACTH: Adrenocorticotropic hormone
DHEA: Dehydroepiandrosterone
HPG: Hypothalamic–pituitary–gonadal
GnRH: Gonadotropin-releasing hormone
LH: Luteinizing hormone
FSH: Follicle stimulating hormone
GPR54: G protein-coupled receptor
ZR: Zona reticularis
ZF: Zona fasciculata
ZG: Zona glomerulosa
DHAS: Dehydroepiandrosterone Sulfate
ELISA: Enzyme-linked immunoassay
WGCNA: Weighted gene co-expression network analysis
PVN: Paraventricular nucleus
CRH: Corticotropin releasing hormone
DEGs: Differentially expressed genes
